# Supplementary material for: Analysis of fibrosis in control or pressure overloaded rat hearts after mechanical unloading by heterotopic heart transplantation
Source: Sci Rep. 2019 Apr 5;9:5710. doi: 10.1038/s41598-019-42263-1 (PMC6451012; doi:10.1038/s41598-019-42263-1)
Supplement: Supplementary file 1 — Supplementary Dataset 1 [file 41598_2019_42263_MOESM1_ESM.pdf]

**Analysis of fibrosis in control or pressure overloaded rat hearts after mechanical unloading by heterotopic heart transplantation**

**Andreas Schaefer[1,2,3,*], MD, MHBA; Yvonne Schneeberger[1,2,3,*], MD, MHBA; Steven Schulz[2,4]; Susanne Krasemann[5], PhD; Tessa Werner[2,4], PhD; Angelika Piasecki[4]; Grit Höppner[4]; Christian Müller[2,6], Karoline Morhenn[2,7], Kristina Lorenz[8], PhD; David Wieczorek[9], PhD; Alexander P Schwoerer[2,3], MD, MME; Thomas Eschenhagen[2,4], MD; Heimo Ehmke[2,3], MD; Hermann Reichenspurner[1,2], MD, PhD; Justus Stenzig[2,4,#], MD, PhD; Friederike Cuello[2,4,#], PhD**

[1]Department of Cardiovascular Surgery, University Heart Center Hamburg; [2]DZHK (German Centre for Cardiovascular Research) partner site Hamburg/Kiel/Lübeck; [3]Department of Cellular and Integrative Physiology, University Medical Center Hamburg-Eppendorf; [4]Department of Experimental Pharmacology and Toxicology, University Medical Center Hamburg-Eppendorf; [5]Institute of Neuropathology, University Medical Center Hamburg-Eppendorf; [6]Department of General and Interventional Cardiology, University Heart Center, Hamburg, Germany; [7]Department of Clinical Pharmacology and Toxicology, University Medical Center Hamburg-Eppendorf; [8]Comprehensive Heart Failure Center, Würzburg; Leibniz-Institut für Analytische Wissenschaften-ISAS-e.V. Dortmund; West German Heart and Vascular Center, Essen; [9]Cardiovascular Center, University of Cincinnati, Ohio, USA

*AS and YS contributed equally to the work; #JS and FC contributed equally to the work

**Total word count: 8344**

**Corresponding author:**
Andreas Schaefer, MD, MHBA
University Heart Center Hamburg, Martinistrasse 52, 20246 Hamburg, Germany
**Tel.:** + 49 40 7410 52440
**Fax.:** +49 040 7410 54931
**Email:** and.schaefer@uke.de

**Supplement**

**Supplementary Table 1. QPCR primer sequences**

| Gene | Sequence |
|---|---|
| Acta1 | AGGACCTGTACGCCAACAAC |
| | ACATCTGCTGGAAGGTGGAC |
| Atp2a2 | GGCTCGTGGGCTCCATCTGC |
| | TCCAGTATTGCAGGCTCCAGGT |
| Col1a1 | TGGACCTCCGGCTCCTGCTC |
| | TCGCACACAGCCGTGCCATT |
| Col3a1 | GGACACAGAGGCTTCGATGG |
| | CTCGAGCACCGTCATTACCC |
| Gusb | CTCGAGCACCGTCATTACCC |
| | CTCGAGCACCGTCATTACCC |
| Myh6 | GGGGCAAGGTCACTGCCGAA |
| | GGCCGCATAGCGCTCCTTGA |
| Myh7 | GGGTATCCGCATCTGTAGGA |
| | TTGGTGTGGCCAAACTTGTA |
| Nppa | CCTCGGAGCCTGCGAAGGTCA |
| | TGTGACACACCGCAAGGGCTTG |
| Nppb | GACGGGCTGAGGTTGTTTTA |
| | ACTGTGGCAAGTTTGTGCTG |

Sequences for the primer pairs used for qPCR based expression analysis

**Supplemental Figure 1. Apoptosis evaluation by cleaved caspase staining**

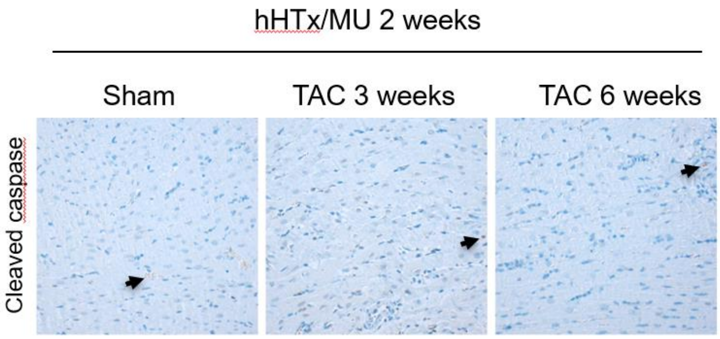


hHTx: Heterotopic heart transplantation; MU: Mechanical unloading

Sections were subjected to caspase staining (arrows pointing at putative apoptotic nuclei)

# Supplementary Table 2. Nanostring raw data

| Gene Name | Accession # | Class Name | 20170119_3 | 20170119_3 | 20170125_3 | 20170125_3 | 20170125_3 | 20170125_3 | 20170125_30101 | 20170125_30101 | 20170125_30101 | 20170125_30101 | 20170125_30101 | 20170125_30101 | 20170125_30101 | 20170125_30101 | 20170119_30101 | 20170119_30101 | 20170119_30101 | 20170119_30101 | 20170119_30101 | 20170119_30101 | 20170119_30101 | 20170119_30101 | 20170119_30101 | 20170119_30101831380717-01_San |
|---|---|---|---|---|---|---|---|---|---|---|---|---|---|---|---|---|---|---|---|---|---|---|---|---|---|---|
| | | | Sham | Sham | Sham | Sham | TAC 3 w | TAC 3 w | TAC 3 week | TAC 3 week | TAC 3 week | TAC 6 week | TAC 6 week | TAC 6 week | TAC 6 week | TAC 6 week | Sham hHTX | Sham hHTX | Sham hHTX | hHTX/MU T | hHTX/MU T | hHTX/MU T | hHTX/MU T | hHTX/MU T | hHTX/MU T | hHTX/MU TAC 6 wk |
| Acta1 | NM_019212 | Endogenous | 67.00 | 43.00 | 224.00 | 357.00 | 313.00 | 164.00 | 300.00 | 885.00 | 435.00 | 870.00 | 481.00 | 613.00 | 1122.00 | 401.00 | 162.00 | 153.00 | 100.00 | 412.00 | 341.00 | 561.00 | 410.00 | 222.00 | 250.00 | 37.00 |
| Acta2 | NM_03100 | Endogenous | 19.00 | 109.00 | 17.00 | 16.00 | 18.00 | 13.00 | 53.00 | 19.00 | 54.00 | 23.00 | 48.00 | 22.00 | 20.00 | 21.00 | 16.00 | 20.00 | 37.00 | 36.00 | 38.00 | 25.00 | 25.00 | 61.00 | 61.00 | 24.00 |
| Actc1 | NM_019183 | Endogenous | 163027.00 | 44230.00 | 265223.00 | 309549.00 | 274478.00 | 266812.00 | 158976.00 | 216298.00 | 171767.00 | 249043.00 | 206030.00 | 256115.00 | 247959.00 | 156352.00 | 187190.00 | 237066.00 | 53730.00 | 204037.00 | 138447.00 | 289155.00 | 175310.00 | 160217.00 | 102724.00 | 95889.00 |
| Actn2 | NM_001170 | Endogenous | 4858.00 | 1599.00 | 5802.00 | 6117.00 | 7189.00 | 5799.00 | 4470.00 | 6147.00 | 3706.00 | 4755.00 | 5317.00 | 4834.00 | 3924.00 | 4464.00 | 6190.00 | 6937.00 | 1677.00 | 4395.00 | 3201.00 | 6819.00 | 5687.00 | 3561.00 | 2719.00 | 4353.00 |
| Atp2a2 | NM_00110 | Endogenous | 499.00 | 131.00 | 718.00 | 780.00 | 758.00 | 798.00 | 490.00 | 461.00 | 486.00 | 477.00 | 501.00 | 611.00 | 430.00 | 429.00 | 448.00 | 645.00 | 167.00 | 588.00 | 312.00 | 677.00 | 336.00 | 303.00 | 242.00 | 435.00 |
| Bax | NM_017059 | Endogenous | 200.00 | 575.00 | 331.00 | 406.00 | 312.00 | 287.00 | 310.00 | 265.00 | 304.00 | 351.00 | 334.00 | 244.00 | 250.00 | 190.00 | 247.00 | 320.00 | 1340.00 | 236.00 | 261.00 | 400.00 | 292.00 | 348.00 | 574.00 | 301.00 |
| Bcl2 | NM_01699 | Endogenous | 126.00 | 154.00 | 207.00 | 203.00 | 243.00 | 182.00 | 194.00 | 176.00 | 152.00 | 194.00 | 207.00 | 173.00 | 163.00 | 161.00 | 233.00 | 327.00 | 319.00 | 166.00 | 136.00 | 240.00 | 245.00 | 180.00 | 285.00 | 218.00 |
| Casp3 | NM_01292 | Endogenous | 113.00 | 368.00 | 177.00 | 198.00 | 163.00 | 148.00 | 198.00 | 129.00 | 147.00 | 201.00 | 247.00 | 142.00 | 99.00 | 80.00 | 153.00 | 210.00 | 1293.00 | 145.00 | 175.00 | 301.00 | 183.00 | 229.00 | 365.00 | 266.00 |
| Casq2 | NM_017131. | Endogenous | 11992.00 | 4065.00 | 13295.00 | 13312.00 | 12328.00 | 16903.00 | 7686.00 | 12534.00 | 9464.00 | 11580.00 | 12679.00 | 13882.00 | 11922.00 | 10552.00 | 13801.00 | 16903.00 | 3484.00 | 11033.00 | 8619.00 | 17674.00 | 12213.00 | 11199.00 | 8283.00 | 9594.00 |
| Cdh5 | NM_001107 | Endogenous | 16.00 | 38.00 | 36.00 | 18.00 | 34.00 | 46.00 | 28.00 | 45.00 | 29.00 | 41.00 | 35.00 | 45.00 | 21.00 | 10.00 | 16.00 | 30.00 | 46.00 | 17.00 | 19.00 | 23.00 | 37.00 | 36.00 | 47.00 | 29.00 |
| Col1a1 | NM_05330 | Endogenous | 4423.00 | 93485.00 | 6282.00 | 6745.00 | 7110.00 | 3332.00 | 10590.00 | 9288.00 | 9254.00 | 6175.00 | 16217.00 | 1934.00 | 3458.00 | 6203.00 | 7888.00 | 8752.00 | 53238.00 | 5291.00 | 5734.00 | 17881.00 | 24256.00 | 51888.00 | 135936.00 | 35024.00 |
| Col3a1 | NM_03208 | Endogenous | 3250.00 | 22175.00 | 6894.00 | 7498.00 | 6642.00 | 4199.00 | 8879.00 | 7193.00 | 8862.00 | 6978.00 | 13067.00 | 2185.00 | 2936.00 | 4091.00 | 6042.00 | 8135.00 | 42295.00 | 4471.00 | 5626.00 | 17352.00 | 13782.00 | 20151.00 | 44898.00 | 20979.00 |
| Ctgf | NM_02226 | Endogenous | 10.00 | 10.00 | 17.00 | 8.00 | 18.00 | 23.00 | 8.00 | 9.00 | 11.00 | 11.00 | 5.00 | 12.00 | 5.00 | 7.00 | 11.00 | 16.00 | 5.00 | 6.00 | 8.00 | 25.00 | 11.00 | 15.00 | 12.00 | 10.00 |
| Fhl1 | NM_14566 | Endogenous | 2191.00 | 2560.00 | 3524.00 | 3972.00 | 3534.00 | 3442.00 | 4843.00 | 5551.00 | 5199.00 | 6539.00 | 6425.00 | 4544.00 | 6965.00 | 3803.00 | 4035.00 | 4551.00 | 2640.00 | 4201.00 | 3983.00 | 9884.00 | 5433.00 | 3905.00 | 7897.00 | 2875.00 |
| Fhl2 | NM_031677 | Endogenous | 19203.00 | 3234.00 | 26149.00 | 28819.00 | 34880.00 | 24040.00 | 8540.00 | 13544.00 | 9974.00 | 15136.00 | 12281.00 | 20129.00 | 12214.00 | 8417.00 | 22527.00 | 25248.00 | 5343.00 | 14895.00 | 6344.00 | 24577.00 | 15432.00 | 14937.00 | 6948.00 | 13031.00 |
| Fn1 | NM_019143 | Endogenous | 106.00 | 3318.00 | 147.00 | 193.00 | 208.00 | 170.00 | 388.00 | 283.00 | 411.00 | 181.00 | 594.00 | 100.00 | 207.00 | 263.00 | 312.00 | 236.00 | 1263.00 | 324.00 | 610.00 | 639.00 | 543.00 | 1849.00 | 3111.00 | 1769.00 |
| Meox1 | NM_001108 | Endogenous | 15.00 | 204.00 | 33.00 | 37.00 | 36.00 | 22.00 | 59.00 | 85.00 | 69.00 | 30.00 | 47.00 | 23.00 | 27.00 | 42.00 | 25.00 | 29.00 | 104.00 | 60.00 | 33.00 | 37.00 | 39.00 | 48.00 | 147.00 | 56.00 |
| Myh6 | NM_01723 | Endogenous | 57434.00 | 8344.00 | 132020.00 | 142936.00 | 169683.00 | 132600.00 | 94462.00 | 74059.00 | 73993.00 | 72074.00 | 90185.00 | 68233.00 | 28548.00 | 77092.00 | 67398.00 | 56386.00 | 2745.00 | 74959.00 | 43894.00 | 86206.00 | 38816.00 | 24616.00 | 14676.00 | 27842.00 |
| Myh7 | NM_01724 | Endogenous | 3025.00 | 1256.00 | 120.00 | 196.00 | 140.00 | 123.00 | 802.00 | 1855.00 | 612.00 | 1295.00 | 796.00 | 1959.00 | 2704.00 | 1133.00 | 5947.00 | 6755.00 | 1533.00 | 1572.00 | 1128.00 | 4482.00 | 5033.00 | 2405.00 | 2857.00 | 4461.00 |
| Nfkb1 | NM_00127 | Endogenous | 195.00 | 243.00 | 182.00 | 183.00 | 153.00 | 138.00 | 171.00 | 124.00 | 152.00 | 177.00 | 211.00 | 119.00 | 136.00 | 95.00 | 171.00 | 269.00 | 1028.00 | 203.00 | 110.00 | 365.00 | 220.00 | 253.00 | 281.00 | 193.00 |
| Nppa | NM_012612 | Endogenous | 4625.00 | 18535.00 | 5294.00 | 12990.00 | 5003.00 | 2710.00 | 294292.00 | 144168.00 | 267413.00 | 153321.00 | 216511.00 | 58945.00 | 197699.00 | 178652.00 | 8713.00 | 26517.00 | 18225.00 | 172823.00 | 323397.00 | 38282.00 | 45832.00 | 28117.00 | 64506.00 | 3387.00 |
| Nppb | NM_031545 | Endogenous | 3794.00 | 3784.00 | 5464.00 | 6799.00 | 3745.00 | 2831.00 | 11076.00 | 15670.00 | 17238.00 | 14802.00 | 11534.00 | 12203.00 | 17476.00 | 11382.00 | 7084.00 | 11759.00 | 3988.00 | 10090.00 | 10380.00 | 15514.00 | 11252.00 | 7054.00 | 9861.00 | 3926.00 |
| Pln | NM_02270 | Endogenous | 29649.00 | 7153.00 | 29162.00 | 28068.00 | 28179.00 | 28186.00 | 9835.00 | 19825.00 | 10295.00 | 19055.00 | 17309.00 | 27171.00 | 19118.00 | 18168.00 | 32343.00 | 37077.00 | 8613.00 | 20294.00 | 6856.00 | 29357.00 | 23933.00 | 20865.00 | 13957.00 | 24223.00 |
| Postn | NM_001108 | Endogenous | 100.00 | 855.00 | 116.00 | 136.00 | 95.00 | 83.00 | 212.00 | 167.00 | 172.00 | 227.00 | 272.00 | 86.00 | 403.00 | 54.00 | 137.00 | 115.00 | 2268.00 | 94.00 | 199.00 | 547.00 | 222.00 | 739.00 | 845.00 | 556.00 |
| Ppp1r1a | NM_02267 | Endogenous | 73.00 | 22.00 | 80.00 | 77.00 | 64.00 | 80.00 | 65.00 | 50.00 | 79.00 | 47.00 | 72.00 | 54.00 | 22.00 | 62.00 | 48.00 | 80.00 | 13.00 | 63.00 | 63.00 | 64.00 | 59.00 | 52.00 | 28.00 | 48.00 |
| Rcan1 | NM_153724 | Endogenous | 226.00 | 139.00 | 471.00 | 653.00 | 391.00 | 227.00 | 469.00 | 576.00 | 531.00 | 959.00 | 519.00 | 951.00 | 771.00 | 313.00 | 607.00 | 854.00 | 340.00 | 662.00 | 587.00 | 1256.00 | 533.00 | 500.00 | 303.00 | 262.00 |
| Ryr2 | NM_03207 | Endogenous | 4777.00 | 2346.00 | 7323.00 | 6877.00 | 10842.00 | 7364.00 | 5613.00 | 7190.00 | 3949.00 | 5556.00 | 5965.00 | 6950.00 | 5727.00 | 6203.00 | 9617.00 | 8751.00 | 1385.00 | 5912.00 | 2828.00 | 8438.00 | 7260.00 | 3081.00 | 4228.00 | 6805.00 |
| S100a4 | NM_012618 | Endogenous | 398.00 | 2464.00 | 290.00 | 313.00 | 283.00 | 288.00 | 621.00 | 713.00 | 514.00 | 610.00 | 1002.00 | 390.00 | 1667.00 | 430.00 | 777.00 | 986.00 | 8757.00 | 544.00 | 990.00 | 1839.00 | 2321.00 | 1944.00 | 2335.00 | 2292.00 |
| Srf | NM_001109 | Endogenous | 529.00 | 569.00 | 1252.00 | 1235.00 | 1183.00 | 641.00 | 895.00 | 824.00 | 1022.00 | 842.00 | 798.00 | 782.00 | 787.00 | 613.00 | 920.00 | 991.00 | 816.00 | 874.00 | 667.00 | 1447.00 | 791.00 | 623.00 | 774.00 | 702.00 |
| Vwf | NM_05388 | Endogenous | 117.00 | 354.00 | 185.00 | 216.00 | 204.00 | 211.00 | 349.00 | 322.00 | 251.00 | 189.00 | 367.00 | 196.00 | 149.00 | 288.00 | 591.00 | 330.00 | 109.00 | 349.00 | 388.00 | 744.00 | 278.00 | 226.00 | 498.00 | 407.00 |
| Abcf1 | NM_001109 | Housekeeping | 341.00 | 329.00 | 814.00 | 859.00 | 840.00 | 518.00 | 614.00 | 716.00 | 560.00 | 827.00 | 660.00 | 760.00 | 649.00 | 488.00 | 600.00 | 641.00 | 649.00 | 672.00 | 457.00 | 747.00 | 484.00 | 407.00 | 462.00 | 438.00 |
| Actb | NM_031144 | Housekeeping | 2059.00 | 14286.00 | 3086.00 | 3520.00 | 4799.00 | 2586.00 | 3344.00 | 3397.00 | 2555.00 | 3015.00 | 3706.00 | 1859.00 | 1988.00 | 2451.00 | 3920.00 | 4101.00 | 44821.00 | 2858.00 | 2489.00 | 5905.00 | 4525.00 | 7021.00 | 11643.00 | 9100.00 |
| Cltc | NM_01929 | Housekeeping | 820.00 | 1772.00 | 1061.00 | 1073.00 | 1161.00 | 916.00 | 914.00 | 967.00 | 890.00 | 914.00 | 1089.00 | 793.00 | 817.00 | 809.00 | 1307.00 | 1459.00 | 2377.00 | 934.00 | 715.00 | 1621.00 | 1403.00 | 1228.00 | 1988.00 | 1383.00 |
| Gapdh | NM_017008 | Housekeeping | 47870.00 | 25799.00 | 90489.00 | 107729.00 | 92176.00 | 91287.00 | 52404.00 | 49267.00 | 54549.00 | 72299.00 | 67171.00 | 70010.00 | 51744.00 | 39691.00 | 65469.00 | 67378.00 | 42504.00 | 57951.00 | 46291.00 | 83157.00 | 42517.00 | 42641.00 | 26162.00 | 34277.00 |
| Pgk1 | NM_05329 | Housekeeping | 38.00 | 40.00 | 58.00 | 53.00 | 30.00 | 45.00 | 43.00 | 61.00 | 47.00 | 38.00 | 46.00 | 61.00 | 50.00 | 40.00 | 33.00 | 41.00 | 50.00 | 46.00 | 34.00 | 64.00 | 36.00 | 48.00 | 36.00 | 36.00 |
| Tubb5 | NM_173102 | Housekeeping | 591.00 | 1734.00 | 677.00 | 687.00 | 694.00 | 572.00 | 652.00 | 823.00 | 629.00 | 762.00 | 901.00 | 422.00 | 563.00 | 474.00 | 562.00 | 730.00 | 3910.00 | 512.00 | 402.00 | 1163.00 | 852.00 | 965.00 | 1190.00 | 814.00 |
| NEG_A | ERCC_000 | Negative | 6.00 | 4.00 | 5.00 | 13.00 | 17.00 | 5.00 | 12.00 | 3.00 | 2.00 | 7.00 | 6.00 | 7.00 | 4.00 | 8.00 | 6.00 | 7.00 | 10.00 | 6.00 | 2.00 | 13.00 | 3.00 | 4.00 | 9.00 | 3.00 |
| NEG_B | ERCC_000 | Negative | 14.00 | 6.00 | 15.00 | 14.00 | 17.00 | 19.00 | 9.00 | 12.00 | 14.00 | 9.00 | 13.00 | 15.00 | 11.00 | 6.00 | 6.00 | 10.00 | 5.00 | 9.00 | 11.00 | 17.00 | 8.00 | 16.00 | 11.00 | 10.00 |
| NEG_C | ERCC_000 | Negative | 43.00 | 9.00 | 74.00 | 97.00 | 90.00 | 108.00 | 66.00 | 60.00 | 71.00 | 77.00 | 56.00 | 73.00 | 57.00 | 37.00 | 38.00 | 86.00 | 20.00 | 51.00 | 50.00 | 87.00 | 45.00 | 49.00 | 23.00 | 39.00 |
| NEG_D | ERCC_000 | Negative | 17.00 | 8.00 | 23.00 | 24.00 | 42.00 | 33.00 | 16.00 | 20.00 | 12.00 | 25.00 | 14.00 | 29.00 | 29.00 | 10.00 | 20.00 | 27.00 | 10.00 | 21.00 | 8.00 | 36.00 | 16.00 | 25.00 | 15.00 | 13.00 |
| NEG_E | ERCC_000 | Negative | 10.00 | 4.00 | 21.00 | 21.00 | 36.00 | 24.00 | 24.00 | 4.00 | 20.00 | 14.00 | 11.00 | 10.00 | 9.00 | 11.00 | 5.00 | 16.00 | 10.00 | 12.00 | 12.00 | 14.00 | 10.00 | 15.00 | 8.00 | 5.00 |
| NEG_F | ERCC_0012 | Negative | 8.00 | 7.00 | 11.00 | 7.00 | 11.00 | 12.00 | 43.00 | 18.00 | 33.00 | 20.00 | 17.00 | 8.00 | 20.00 | 15.00 | 8.00 | 15.00 | 20.00 | 22.00 | 21.00 | 11.00 | 10.00 | 9.00 | 11.00 | 8.00 |
| POS_A | ERCC_000 | Positive | 42433.00 | 31713.00 | 39323.00 | 37441.00 | 50120.00 | 53228.00 | 26898.00 | 45464.00 | 32935.00 | 40879.00 | 32331.00 | 45853.00 | 40114.00 | 36623.00 | 40795.00 | 51931.00 | 62844.00 | 35590.00 | 19514.00 | 46232.00 | 44487.00 | 50611.00 | 54999.00 | 56468.00 |
| POS_B | ERCC_0011 | Positive | 7486.00 | 6258.00 | 6649.00 | 6217.00 | 8322.00 | 8945.00 | 4538.00 | 7799.00 | 5463.00 | 6892.00 | 5553.00 | 7651.00 | 7031.00 | 6561.00 | 7521.00 | 8603.00 | 10974.00 | 6137.00 | 3550.00 | 7677.00 | 7787.00 | 8780.00 | 9579.00 | 9555.00 |
| POS_C | ERCC_000 | Positive | 2912.00 | 2748.00 | 2560.00 | 2296.00 | 3049.00 | 3353.00 | 1559.00 | 2900.00 | 1870.00 | 2508.00 | 2003.00 | 2991.00 | 2826.00 | 2574.00 | 2824.00 | 3160.00 | 4171.00 | 2289.00 | 1078.00 | 2800.00 | 3000.00 | 3403.00 | 3635.00 | 3601.00 |
| POS_D | ERCC_000 | Positive | 347.00 | 329.00 | 260.00 | 249.00 | 362.00 | 393.00 | 203.00 | 358.00 | 239.00 | 291.00 | 220.00 | 357.00 | 318.00 | 284.00 | 307.00 | 357.00 | 511.00 | 256.00 | 133.00 | 332.00 | 321.00 | 421.00 | 495.00 | 456.00 |
| POS_E | ERCC_000 | Positive | 145.00 | 99.00 | 139.00 | 132.00 | 170.00 | 170.00 | 95.00 | 153.00 | 120.00 | 157.00 | 117.00 | 132.00 | 114.00 | 106.00 | 130.00 | 153.00 | 218.00 | 111.00 | 69.00 | 152.00 | 154.00 | 151.00 | 200.00 | 178.00 |
| POS_F | ERCC_0011 | Positive | 27.00 | 42.00 | 49.00 | 40.00 | 65.00 | 72.00 | 35.00 | 51.00 | 42.00 | 45.00 | 48.00 | 60.00 | 38.00 | 24.00 | 32.00 | 42.00 | 57.00 | 31.00 | 23.00 | 27.00 | 42.00 | 52.00 | 77.00 | 55.00 |

**Supplemental Figure 2. Raw data / full western immunoblots for figure 6**

pSer282 cMyBP-C

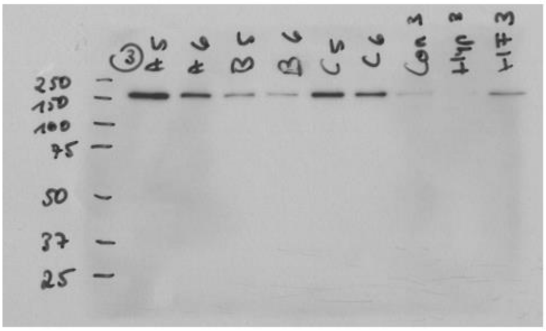

cMyBP-C

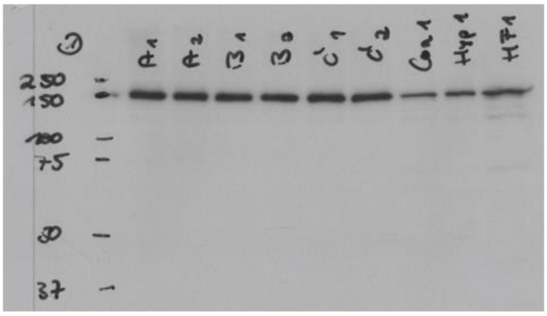

pSer16 PLN

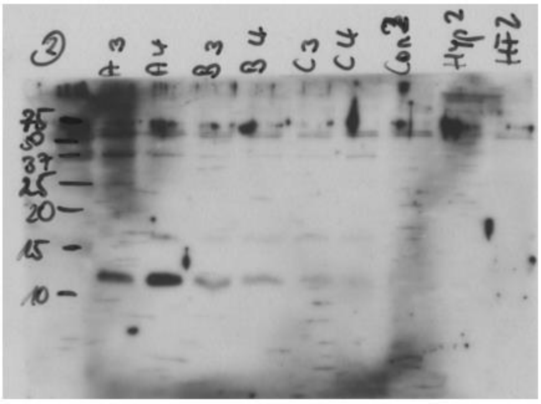

PLN

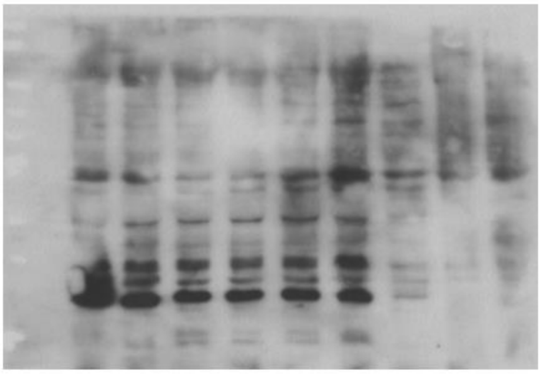

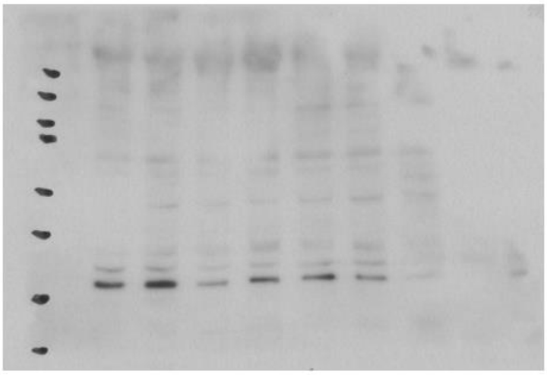

pSer22/23 cTnI

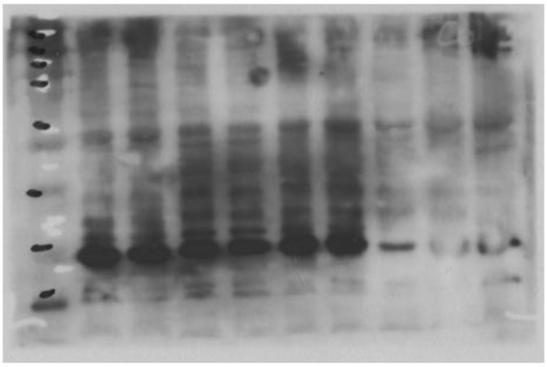

cTnI

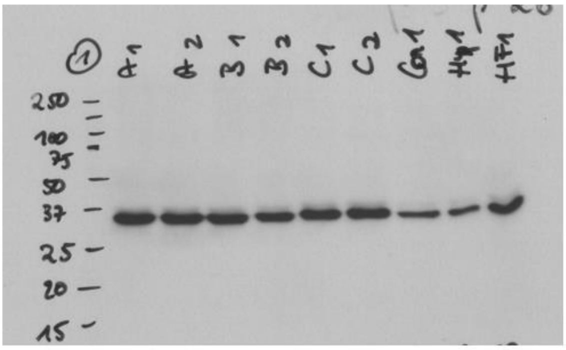


pSer283 TM

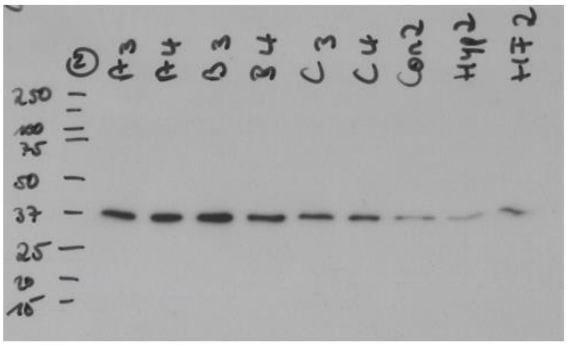


TM

pThr202/Tyr204ERK

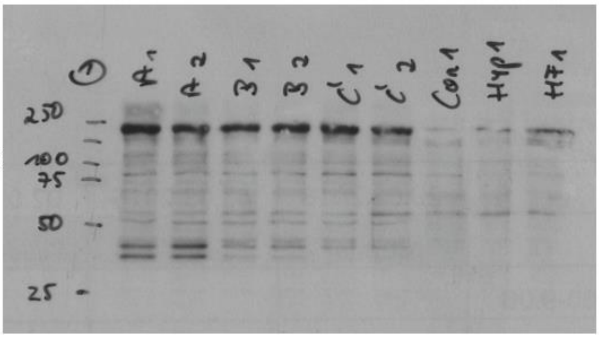

tERK

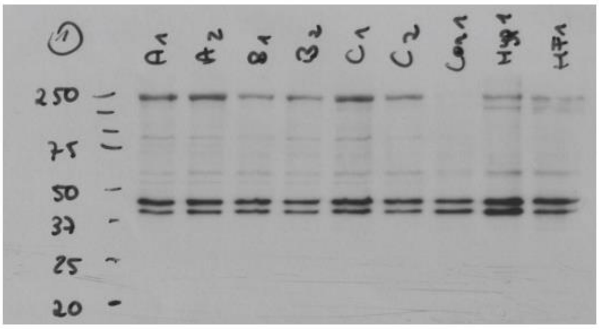

50 kDa

37 kDa

pThr188 ERK

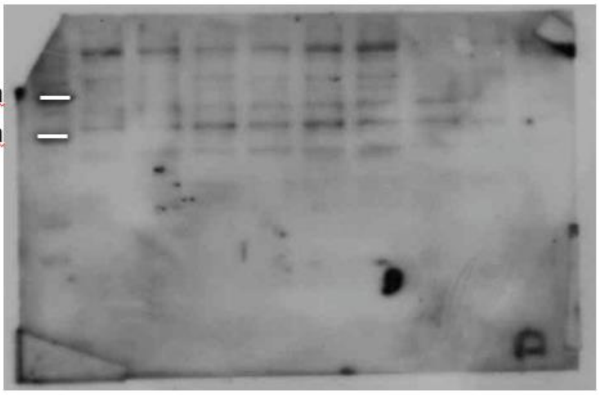

ERK

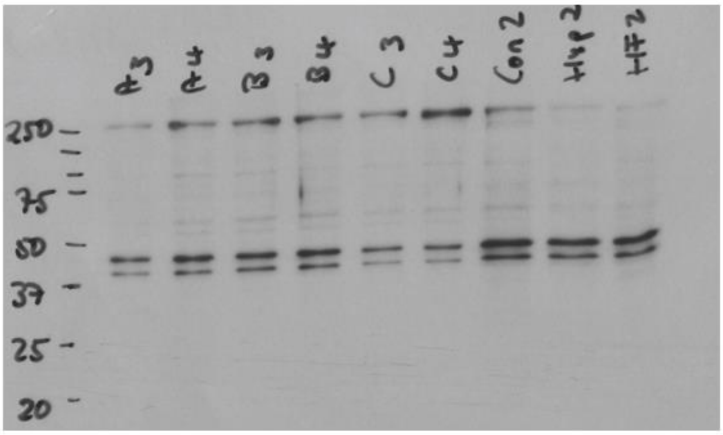

FHL1

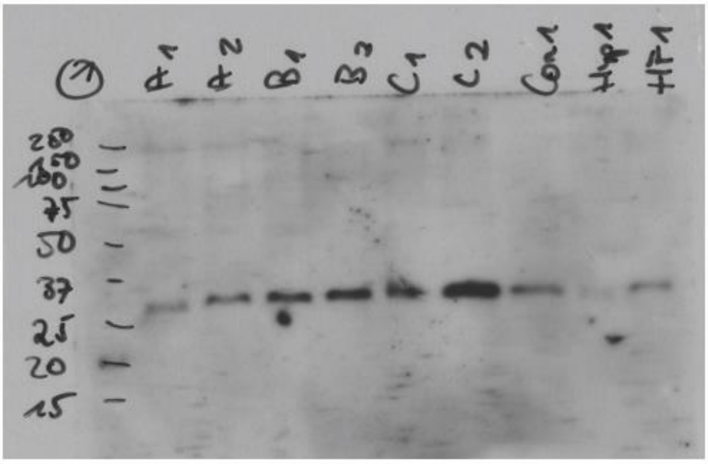
①
A1 A2 B1 B3 C1 C2 Con1 Hyp1 HF1
250
150
100
75
50
37
25
20
15

FHL2

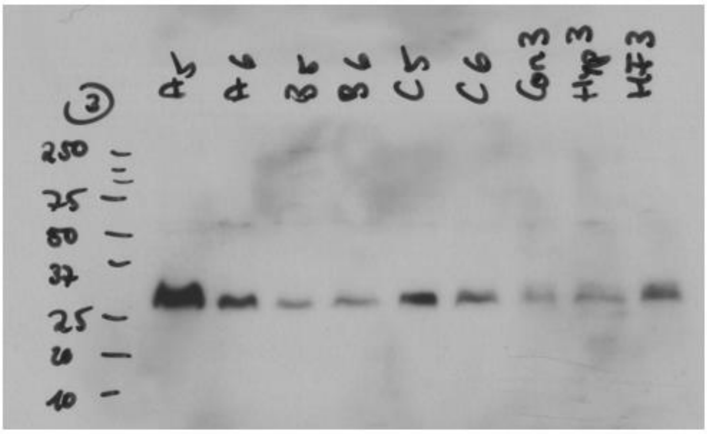
③
A5 A6 B5 B6 C5 C6 Con3 Hyp3 HF3
250
75
50
37
25
20
10
